# Biological Responses of Three-Dimensional Cultured Fibroblasts by Sustained Compressive Loading Include Apoptosis and Survival Activity

**DOI:** 10.1371/journal.pone.0104676

**Published:** 2014-08-07

**Authors:** Toshiki Kanazawa, Gojiro Nakagami, Takeo Minematsu, Takumi Yamane, Lijuan Huang, Yuko Mugita, Hiroshi Noguchi, Taketoshi Mori, Hiromi Sanada

**Affiliations:** 1 Department of Gerontological Nursing/Wound Care Management, Graduate School of Medicine, The University of Tokyo, Tokyo, Japan; 2 Department of Nutritional Sciences, Faculty of Applied Bioscience, Tokyo University of Agriculture, Tokyo, Japan; 3 Department of Life Support Technology, Graduate School of Medicine, The University of Tokyo, Tokyo, Japan; University Hospital Hamburg-Eppendorf, Germany

## Abstract

Pressure ulcers are characterized by chronicity, which results in delayed wound healing due to pressure. Early intervention for preventing delayed healing due to pressure requires a prediction method. However, no study has reported the prediction of delayed healing due to pressure. Therefore, this study focused on biological response-based molecular markers for the establishment of an assessment technology to predict delayed healing due to pressure. We tested the hypothesis that sustained compressive loading applied to three dimensional cultured fibroblasts leads to upregulation of heat shock proteins (HSPs), CD44, hyaluronan synthase 2 (HAS2), and cyclooxygenase 2 (COX2) along with apoptosis *via* disruption of adhesion. First, sustained compressive loading was applied to fibroblast-seeded collagen sponges. Following this, collagen sponge samples and culture supernatants were collected for apoptosis and proliferation assays, gene expression analysis, immunocytochemistry, and quantification of secreted substances induced by upregulation of mRNA and protein level. Compared to the control, the compressed samples demonstrated that apoptosis was induced in a time- and load- dependent manner; vinculin and stress fiber were scarce; HSP90α, CD44, HAS2, and COX2 expression was upregulated; and the concentrations of HSP90α, hyaluronan (HA), and prostaglandin E_2_ (PGE_2_) were increased. In addition, the gene expression of antiapoptotic *Bcl2* was significantly increased in the compressed samples compared to the control. These results suggest that compressive loading induces not only apoptosis but also survival activity. These observations support that HSP90α, HA, and, PGE_2_ could be potential molecular markers for prediction of delayed wound healing due to pressure.

## Introduction

A recent study indicates that the prevalence of pressure ulcer (PU) is 13.7% in all care settings, including acute, long-term, rehabilitation, and home care settings [1]. This unacceptably high prevalence may be related to its chronicity, representing delayed wound healing due to pressure, which mainly inhibits tissue granulation in the wound healing process. A PU is continuously exposed to pressure as noted in its definition that PU is a localized damage to the skin and the underlying tissue, mainly caused by continuous exposure to pressure [2]. This is particularly true in immobile elderly and spinal cord injury patients; thus, it is quite difficult to completely eliminate pressure.

Early intervention for preventing delayed healing of PUs due to pressure requires a prediction method. Although clinical manifestations such as “thickened edges” [3] and “double erythema” [4] have been reported, they only indicate that pressure has already affected the PU healing process and do not help determine an appropriate preventive strategy for detection. To our knowledge, although some studies about delayed wound healing related with malnutrition or infection has reported [5], no study has reported the prediction of delayed wound healing due to pressure.

Why is no method available for predicting delayed healing due to pressure? This could be due to 2 reasons. First, it is quite difficult to estimate pressure-induced mechanical stress within the tissue, which directly causes cell damage and is measured using a pressure sensor such as a multi-pad type device that is widely used in clinical practice [6]. Second, we cannot estimate the magnitude of mechanical stress responsible for cell damage. Even if mechanical stress can be measured, the cellular response that leads to tissue damage is not uniform because of interpatient variability related to comorbidity, wound location, nutrition, and age [5], [7]–[9]. We therefore considered that analysis of the cellular response to mechanical stress is the best approach for the prediction of delayed wound healing due to pressure. To investigate the cellular response, it is very important to reveal the molecular-level phenomena within the cell that lead to cell damage; thus, an *in vitro* model would be the most suitable option for this purpose.

In the present study, we focused on the biological response-based molecular markers for the establishment of an effective assessment technology to predict delayed wound healing due to pressure. Specifically, we investigated the changes in gene expression by applying sustained compressive loading to the fibroblasts in a collagen sponge, which mimics the situation when pressure is continuously applied to the granulation tissue filled with fibroblasts and extracellular matrix (ECM). We subsequently identified the secreted substance along with gene expression as a molecular marker that could be collected noninvasively from the wound exudates in a clinical setting. Moseley et al. [8] reported in their review that analysis of wound exudates has a scientific and objective rationale for assessing the wound condition.

Although there are few studies that applied sustained compressive loading to the fibroblasts under three-dimensional (3D) culture for this purpose, in exploring the molecular markers we decided to investigate the gene expression of *heat shock proteins* (*Hsps*), *Cd44*, *hyaluronan synthase 2* (*Has2*), and *cyclooxygenase 2* (*Cox2)* as key factors related with mechanical stress and apoptosis [10]–[17]. In addition, our study focused on apoptotic cell death triggered by loss of ECM contacts, which indicates disruption of cell adhesion [13], [14], [18]. Previous studies have reported that increased apoptosis within the granulation tissue may contribute to impaired wound healing [19], and mechanical stress may induce apoptosis via disruption of adhesion [20], [21], which leads to the idea that compression induces apoptosis triggered by the disruption of adhesion.

To test the hypothesis that sustained compressive loading applied to 3D cultured fibroblasts leads to upregulation of HSPs, CD44, HAS2, and COX2 along with apoptosis *via* disruption of adhesion, we applied sustained compressive loading to fibroblast-seeded collagen sponges.

## Materials and Methods

### Cell culture

The rat fibroblast cell line Rat-1 (RIKEN BioResource Center, Ibaraki, Japan) was grown at 37°C under 5% CO_2_ in DMEM (Nacalai Tesque, Kyoto, Japan) supplemented with 10% FBS (Biowest, Nuaillé, France) and antibiotics (100 U/ml penicillin, 100 µg/ml streptomycin; Nacalai Tesque), and then cultured in a monolayer.

### Cell seeding to the collagen scaffold

A porous atelocollagen sponge (MIGHTY; KOKEN, Tokyo, Japan) was used as a scaffold. The pore size was designed to be 100–200 µm, and the pores were interconnected. Trypsinized cells (2.0×10^7^/pellet) collected by centrifugal force were suspended in 2.4 ml of 0.5% atelocollagen solution (KOKEN) supplemented with 0.3 ml of 10× concentrated DMEM prepared by dissolving DMEM powder (Biological Industries, Beit Haemek, Israel) in sterilized distilled water and 0.3 ml reconstitution buffer (Nitta Gelatin, Osaka, Japan) on ice to produce a cell suspension in a 0.4% collagen solution of DMEM. In total, 100 µl of cell suspension was seeded onto a collagen scaffold (5-mm diameter, 3-mm thick) by centrifugation (500×*g*, 5 min).

### Compressive loading and loading protocol

**Figure 1:**
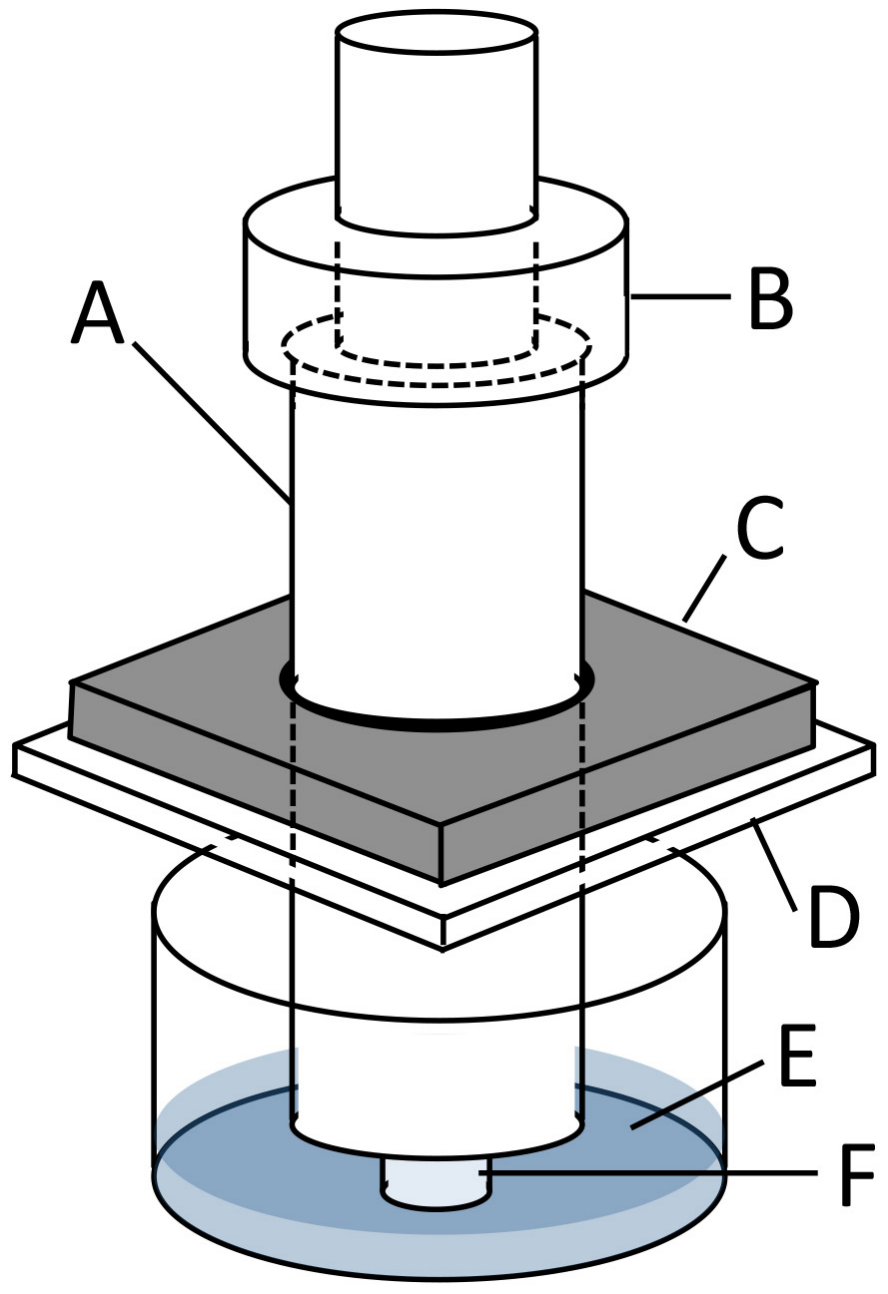
Loading apparatus to apply sustained compressive loading to cells seeded-collagen sponge. In this representation A indicates an indenter (diameter: 10 mm), B the weights, C a stainless steel plate (thick: 5 mm), D a 12-well plate lid, E culture medium, and F a fibroblast-seeded collagen sponge sample (diameter: 5 mm and thick: 3 mm).

Collagen sponge samples were precultured for 24 h and subjected to sustained compressive loading under 5% CO_2_ at 37°C by using a custom-built loading apparatus (Fig. 1). The loading apparatus applied compression to the samples in a 12-well plate with stainless steel indenters, using a 5-mm thick stainless steel plate on top of the 12-well plate to stabilize the indenter. Various weights can be placed on top of these indenters to apply specified compression to the samples. Pressures of 0, 50, 100, or 200 mmHg, by following the report of Swain [22], were applied on samples for 2, 4, or 6 h. The sample with no treatment (0 h–0 mmHg) was analyzed as the baseline. Experiments were repeated 5 times.

### WST-1 assay

Proliferative activity of fibroblasts was analyzed using the colorimetric WST-1 assay (Roche Diagnostics, Basel, Switzerland). In brief, collagen sponge samples after loading were transferred to new 12-well plates in 1 ml of medium containing 100 µl WST-1 reagent per well and then incubated for 1.5 h. The absorbance at 450 nm was measured using a microplate reader, DTX800 (Beckman Coulter, Brea, CA). The proliferative activity of each sample was shown as a relative value of absorbance compared to the baseline sample.

### The terminal deoxy-nucleotidyl transferase-mediated deoxyuridine triphosphate nick end-labeling (TUNEL) staining

Quantification of apoptosis was measured using the *In situ* Apoptosis Detection Kit (TAKARA Bio Inc., Shiga, Japan). One side of each collagen sponge cut in half after the WST-1 assay was fixed in 4% paraformaldehyde in phosphate buffer, dehydrated with series of ethanol, cleansed with series of xylene and embedded in paraffin. Longitudinal 4-µm thick sections were deparaffinized. Following TUNEL staining performed according to the manufacturer’s instruction, the nucleus was stained with DAPI. The stained cells were observed under an inverted fluorescence microscope (DMI4000B; Leica, Wetzlar, Germany). The number of TUNEL-positive cells was counted in 5 fields in the central area of the collagen sponge (magnification ×10), and the proportion of positive cells to total cells was calculated.

### Morphology and immunocytochemistry

Longitudinal 5-µm thick sections of 3D cell culture were deparaffinized and hematoxylin and eosin (H&E) staining was performed.

Rhodamine phaloidin and vinculin staining was performed on 3D cell culture samples as follows. The sections were incubated with rhodamine phaloidin for 45 minutes at room temperature (100 nM in 1% BSA; Cytoskeleton, Denver, CO). For vinculin, the sections were incubated with anti-vinculin rabbit polyclonal antibody (Sigma-Aldrich, St. Louis, MO; diluted 1∶100) for 60 min at room temperature after antigen retrieval (semi boiling for 10 min in 10 mM citrate buffer, pH 6.0). Subsequently, the sections were incubated with Dylight^®^ 488 anti-rabbit IgG antibody (Vector Laboratories, Burlingame, CA; diluted 1∶1000) for 30 min at room temperature. The nucleus was stained with DAPI in both stainings. Between each step, the sections were washed 3 times with PBS for 5 min each. The stained cells were observed under an inverted fluorescence microscope (DMI4000B).

HSP90α, CD44, and COX2 immunostaining was performed on 3D cell culture as follows: the sections were incubated with anti-HSP90α rabbit polyclonal antibody (Lab Vision Corporation, Fremont, CA), anti-HCAM rabbit polyclonal antibody (Santa Cruz Biotechnology, Dallas, TX), or anti-COX2 rabbit monoclonal antibody (Cell Signaling Technology, Danvers, MA) at room temperature for 60 min (each antibody was diluted 1∶50) after quenching of endogenous peroxidase and antigen retrieval (3D cell culture samples were subjected to semi boiling for 10 min). Subsequently, the sections were incubated with biotin-conjugated anti-rabbit IgG antibody (Jackson ImmunoResearch Laboratories, West Grove, PA; diluted 1∶1000) for 30 min at room temperature. Immunoreactions were detected using a VectaStain ABC Kit (Vector Laboratories) with 3,3′- diaminobenzidine tetrahydrochloride substrate (Nacalai Tesque) and counterstained using hematoxylin.

HAS2 immunostaining was performed with anti-HAS2 mouse monoclonal antibody as the primary antibody (Santa Cruz Biotechnology; diluted 1∶50) and then, as the secondary antibody, HRP-conjugated anti-mouse IgG antibody (Bethyl Laboratories, Montgomery, TX; diluted 1∶1000). Any other kind of immunostaining methods was performed as well as methods described above. The samples were observed using an upright microscope (BX41; Olympus, Tokyo, Japan).

### RNA extraction and real-time reverse transcription-polymerase chain reaction (RT-PCR)

Total RNA was extracted from the fibroblasts seeded on a collagen sponge following standard procedures using the RNeasy Plus Mini Kit (QIAGEN, Hilden, Germany). The other side of each collagen sponge, cut in half after the WST-1 assay, was minced and homogenized in liquid nitrogen. cDNA synthesis was performed using the TM100™ Thermal Cycler (Bio-Rad, Richmond, CA) and the High Capacity cDNA Reverse Transcription Kit (Life Technologies, Carlsbad, CA). For quantitative PCR, amplification of the target-specific region of cDNA was performed using Power SYBR^®^ Green PCR Master Mix (Life Technologies) in a real-time PCR system (Mx3000P QPCR System; Agilent Technologies, Santa Clara, CA). The PCR protocol was as follows: 40 cycles at 95°C for 30 s and 60°C for 1 min after preheating at 95°C for 10 min. The expression of the target genes in the 6 h–200 mmHg group relative to the value in the 6 h–0 mmHg group was calculated by the comparative Ct method using the 18S ribosomal RNA gene as an internal control. The primer sequences are shown in Table 1. We confirmed that WST-1 measurement did not affect the gene expression analysis.

**Table 1:** Primer sequences used for the quantification of gene expression.

Target gene	Primer sequeces (5′-3′)		GenBank
	Forward	Reverse	accession number
*Hsf1*	TTGACTCCATCCTTCGAGA	CCAGGTGATCACTTAGCTC	NM_024393.1
*Hsf2*	TCAGGAAGACAGTTTAGCAT	AAAGGCAGTGTACTGGATAA	NM_031694.2
*Hsp32*	AGTTCAAACAGCTCTATCGT	GTAGTATCTTGAACCAGGCT	NM_012580.2
*Hsp40*	GCGAGATTTTCGACCGCTAT	GATTCCTGCCACCGAAGAAC	NM_001108441.1
*Hsp47*	CTCGTTAATGCCATGTTCTT	TCTCGTCGTCATAGTAGTTG	NM_017173.1
*Hsp60*	TCGCCAGATGAGACCAGTGT	TGGGACTTCCCCAACTCTGT	NM_022229.2
*Hspa5*	CATTCAAGGTGGTTGAAAAG	TGCATCATTGAAGTAAGCTG	NM_013083.2
*Hsc70*	TGAGAATGTTCAGGATTTGC	CATACACCTGGATGAGTACA	NM_024351.2
*Hsp90aa1*	GTGCGGTTAGTCACGTT	TCGAGTAGAAAGTGTTGATG	NM_175761.2
*Bcl2*	GCGTCAACAGGGAGATGTCA	GCTGAGCAGCGTCTTCAGAG	NM_016993.1
*Bax*	GATGATTGCTGACGTGGACA	TGATCAGCTCGGGCACTTTA	NM_017059.2
*Cd44*	CCGTTACGCAGGTGTATTCC	TGTTGAAAGCCTCGCAGAG	NM_012924.2
*Has1*	TTCAAGGCACTGGGTGACTC	CCCAGTATCGAAGGCTGCTC	NM_172323.1
*Has2*	AGGGGACCTGGTGAGACAGA	GGGTCAAGCATGGTGTCTGA	NM_013153.1
*Has3*	GTGTTCGAGCTGTGGTGTGG	GGGGATCTTCCTCCAAGACC	NM_172319.1
*Vcan*	TGAATGTCACTCTAACCCTT	ATTGCCCTTGGAATTTGTG	NM_053663.1
*Tnfaip6*	GCTTTGTAGGAAGATACTGC	CCTTGATTGGATTTAGGTGC	NM_053382.1
*Hyal1*	CCTTCAGTCCTGAGGTTTCC	CCAGTGAGTGTCTGCATTCC	NM_207616.1
*Hyal2*	CAGAACTTAGCCAGATGGAC	CACATTGACTATGTAGGGGA	NM_172040.2
*Hyal3*	TCTTCCCTAGCATCTACCTC	TAGGTCATCCAGAGACAAGA	NM_207599.2
*Mmp2*	ACAGGACCCTGGAGCTTTGA	CTTGCAGATCTCGGGAGTGA	NM_031054.2
*Mmp3*	AAGATGCTGGCATGGAGGTT	TTCGAGTCCAGCTTCCCTGT	NM_133523.2
*Mmp9*	GCGCTGGGCTTAGATCATTC	TGGGACACATAGTGGGAGGA	NM_031055.1
*Mmp13*	ATGTGGAGTGCCTGATGTGG	GCCATCATGGATCCTGGTAAA	NM_133530.1
*Cox2*	CCCACTTCAAGGGAGTCTGG	GCAGTCATCAGCCACAGGAG	NM_017232.3

### ELISA

The culture medium was collected after compressive loading. HSP90α concentration was measured using the Rat Heat Shock Protein 90α ELISA kit (CUSABIO BIOTECH, Wuhan, China). The concentration of hyaluronan (HA) was measured using the QnE Hyaluronic Acid ELISA Assay (Biotech Trading Partners, Encinitas, CA). The concentration of prostaglandin E_2_ (PGE_2_) was measured using the PGE_2_ high sensitivity EIA kit (Enzo Life Sciences, Farmingdale, NY). Each experiment was performed according to the manufacturer’s instructions. The values were normalized based on the cell number measured with the value of the WST-1 assay.

### Statistical analysis

The results have been presented as mean ± SEM value. Statistical differences between the 2 groups were determined using the Student’s t-test. The differences among multiple groups were compared by Dunnett’s method using the baseline or 0 mmHg group as the control. A *p* value <0.05 was considered statistically significant. The software IBM SPSS Statistics for Windows version 20.0 (IBM, Armonk, NY) was used for all statistical analyses.

## Results

### 3. 1. Sustained compressive loading did not induce apparent cell proliferation and induced apoptosis through disruption of adhesion

**Figure 2:**
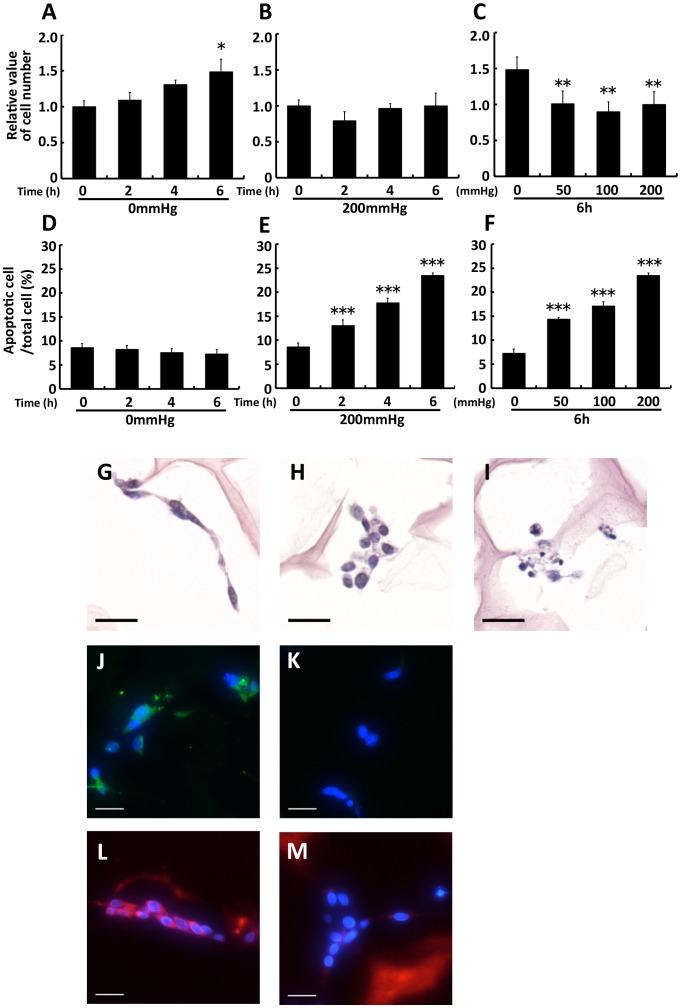
Sustained compressive loading did not induce apparent cell proliferation and induced apoptosis through disruption of adhesion. Fibroblasts were seeded to collagen sponge and incubated for 24: Collagen sponge samples after loading were transferred to new 12-well plates in 1 ml medium containing 100 µl WST-1 reagent per well, and then incubated for 1.5 h. The absorbance of 450 nm was measured. The cell number was shown relative to base line. The results are represented as the mean ± SEM (error bars) of five experiments. D, E, and F: Collagen sponge samples were fixed, dehydrated, cleared, and processed for embedding in paraffin after loading experiments. Sections were prepared at 4-µm thick. Apoptosis assay were performed by TUNEL stain using tissue slides. The number of TUNEL-positive cells was counted in 5 fields in the central area of the collagen sponge (magnification ×10), and the proportion of positive cells to total cells was calculated. The results are represented as the mean ± SEM (error bars) of five experiments. Statistical analysis was performed using the Dunnett’s multiple test: between 2, 4, or 6 h group and 0 h group (A, B, D, and E) or between each of loaded group and nonloaded group (C and F). Statistical significance was taken as *p*<0.05. A value of *p* was expressed as: *; *p*<0.05, **; *p*<0.01, and ***; *p*<0.001. G, H, and I: H&E staining for confirming cell morphology. Collagen sponge samples were prepared as aforementioned. Sections were prepared at 5-mm thick. The distinctive cell morphology observed in the 6 h–0 mmHg group was spindle-shaped cells (G), whereas that in the 6 h–200 mmHg group was nonspindle-shaped cells (H) along with apoptotic bodies (I). J and K: Immunostaining for the FA structural protein vinculin (green). Nucleus stained by DAPI (blue). Vinculin expression was observed in the 6 h–0 mmHg group (J), whereas it was scarcely observed in the 6 h–200 mmHg group (K). L and M: Immunostaining for actin stress fibers by phaloidin (red). Nucleus stained by DAPI (blue). Actin stress fibers were observed in the 6 h–0 mmHg group (L), but not in the 6 h–200 mmHg group (M). Scale bars = 20 µm for all images.

WST-1 assay and TUNEL staining were used to investigate the proliferative and the apoptotic effects of sustained compressive loading at various loading times and intensities in 3D cultured fibroblasts. While the cell number in nonloaded groups significantly increased compared with the baseline in a time-dependent manner (2, 4, and 6 h groups; *p* = 0.872,  = 0.147, and  = 0.018, respectively; Fig. 2A), such an increase over the baseline was not observed in 2, 4, and 6 h–200 mmHg groups (each group; *p*>0.05; Fig. 2B). Moreover, 6-h compressive loading induced a significant reduction in the cell number of the 50, 100, and 200 mmHg groups compared with the 0 mmHg group (each group; *p*<0.01; Fig. 2C). An increase in apoptosis was not observed in nonloaded groups (each group; *p*>0.05; Fig. 2D). In contrast, compressive loading significantly induced apoptosis in a time- and load-dependent manner (according to loading time for 2, 4, and 6 h–200 mmHg, *p* = 0.002, <0.001, and <0.001, respectively; according to loading intensity at 6 h–50, 100, and 200 mmHg, *p*<0.001, <0.001, and <0.001, respectively; Fig. 2E and 2F). Although these results suggest that proliferation and apoptosis occur simultaneously during compressive loading, apparent cell proliferation did not occur. We thus considered this system as the inhibitory state of granulation.

The detachment of anchorage-dependent cells, such as fibroblasts, induces apoptosis which is called “anoikis” [23]. In the process of anoikis, cell detachment from ECM induces apoptosis by disrupting survival signals generated through cytoskeletal rearrangements induced by cell integrin-ECM interactions, including the formation of focal adhesions (FAs) and actin filament stress fibers [18], [24], [25]. We therefore investigated the effects of sustained compressive loading on cell morphology, FA, and actin stress fiber formation in 3D cultured fibroblasts. First, we compared the cell morphology, identified by H&E staining, for the 6 h–0 mmHg and the 6 h–200 mmHg groups. The distinctive cell morphology observed in the 6 h–0 mmHg group was spindle-shaped cells, whereas that in the 6 h–200 mmHg group was nonspindle-shaped cells along with apoptotic bodies (Fig. 2G, 2H, and 2I). Second, we compared the expression of FAs, identified by staining of the FA structural protein vinculin, for the 6 h–0 mmHg and the 6 h–200 mmHg groups. Vinculin expression was observed in the 6 h–0 mmHg group, whereas it was scarcely observed in the 6 h–200 mmHg group (Fig. 2J and 2K). Similarly, actin stress fibers were observed in the 6 h–0 mmHg group, but not in the 6 h–200 mmHg (Fig. 2L and 2M). Thus, in the cells of 6 h–200 mmHg group without apoptotic bodies, vinculin and actin stress fibers were scarce even before the cells underwent apoptosis. The observation that vinculin and actin stress fibers were scarce even in cells without apoptotic bodies supports the proapoptotic effects of compressive loading on fibroblasts by disruption of adhesion.

Gene expression was compared between the 6 h–0 mmHg and the 6 h–200 mmHg groups to narrow down the candidates of the molecular markers because, by integrating the results of cell number and apoptosis, most differences would be observed between these 2 groups. After investigating gene expression, we measured the concentration of secreted substances in the cultured medium of the 0, 50, 100, and 200 mmHg groups to confirm the clinical applicability of markers for predicting tissue damage caused by the compressive loading.

### Stress- and apoptosis-related gene expression was stimulated by 6-h compressive loading

**Figure 3:**
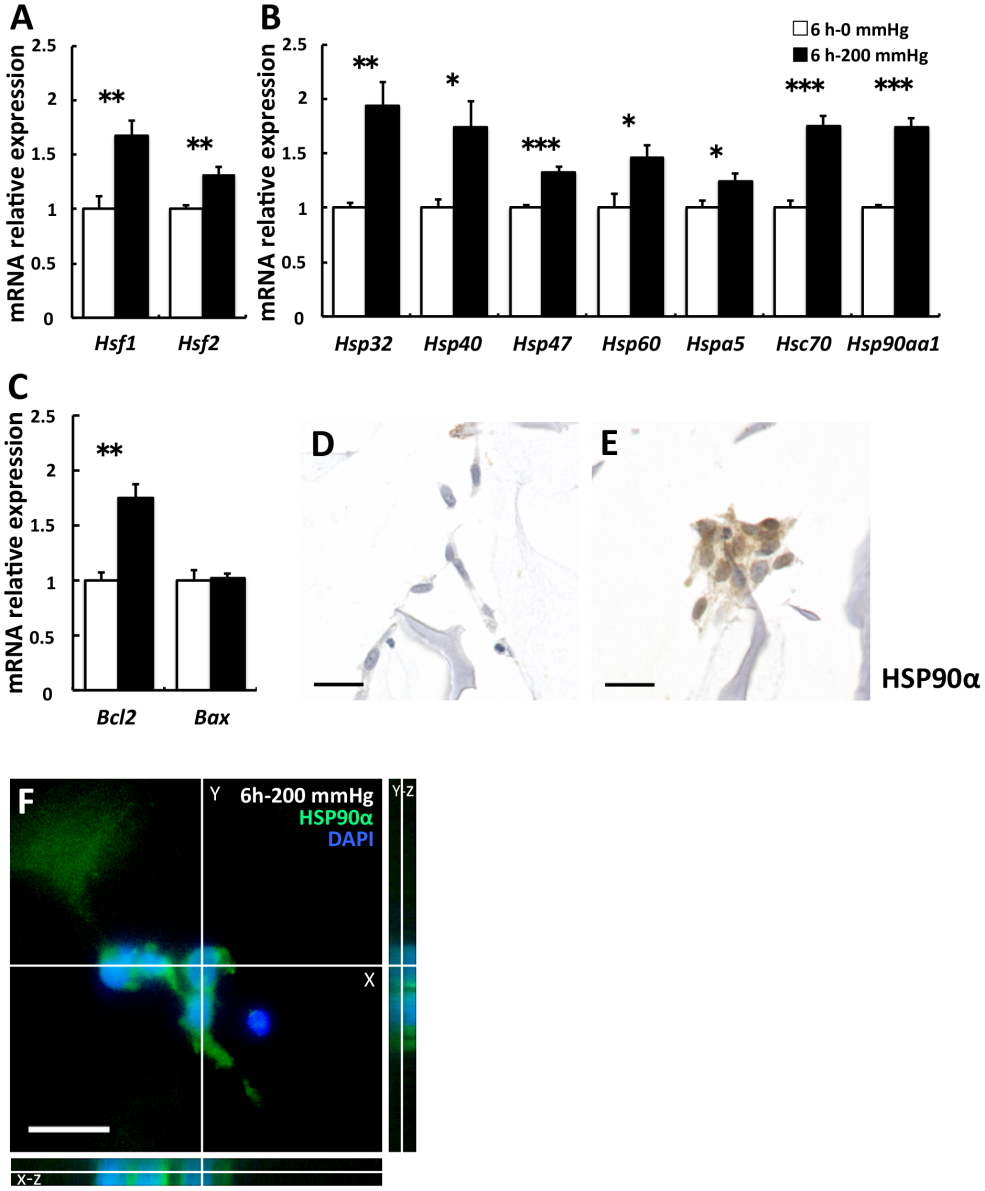
Stress- and apoptosis-related gene expression was stimulated by 6-h compressive loading. Fibroblasts were seeded to collagen sponge and incubated for 24(□) or 200 mmHg (▪) compression for 6 h. Total mRNA was extracted after WST-1 assay, and mRNA expression was assessed using real-time RT-PCR. The expression of the target genes in the 6 h–200 mmHg group relative to the value in the 6 h–0 mmHg group was calculated by the comparative Ct method using the 18S ribosomal RNA gene as an internal control. The results are represented as the mean ± SEM (error bars) of five experiments. Statistical analysis was performed using the Student’s t test between the 0 mmHg group and the 200 mmHg group, and statistical significance was taken as *p*<0.05. A value of *p* was expressed as: *; *p*<0.05, **; *p*<0.01, and ***; *p*<0.001. A: The transcription factors of various *Hsps*. B: various *Hsps* C: *Bcl2* is an antiapoptotic gene, and *Bax* is a proapoptotic gene. D and E: Immunostaining for HSP90α. Representative sections of (D) the 6 h–0 mmHg group and (E) the 6 h–200 mmHg group. Higher expression and nucleus translocation of HSP90α was observed in the 200 mmHg group (E) when compared with the 0 mmHg group (D). Scale bars = 20 µm for all images.

A significant increase in the expression of *heat shock transcription factor 1* (*Hsf1*) and *Hsf2* was observed in the 200 mmHg group compared with the 0 mmHg group *(Hsf1* and *Hsf2*; *p* = 0.006 and  = 0.004, respectively; Fig. 3A). Expression of these genes is induced by the disruption of adhesion [26], and HSF1 and HSF2 bind to the regulatory site of various *Hsp* genes. Following this, we investigated the influence of compressive loading on the gene expression of various HSPs, which are stress-responsive proteins against mechanical stress, elevated temperature, hypoxia, lowered pH, and reactive oxygen species (ROS) [27]. The expression of various *Hsps* was significantly higher in the 200 mmHg group than in the 0 mmHg group (*Hsp32*, *Hsp40*, *Hsp47*, *Hsp60*, *Hspa5*, *Hsc70*, and *Hsp90aa1*; *p* = 0.002,  = 0.019, <0.001,  = 0.024,  = 0.033, <0.001, and <0.001, respectively; Fig. 3B). Upregulation of various *Hsps* indicates that stress responses by compressive loading occurred in fibroblasts. To examine the condition of nonapoptotic cells, we investigated the expression of antiapoptotic *Bcl2* [28] and proapoptotic *Bax* [29]. The results indicated that *Bcl2* levels were significantly higher in the 200 mmHg group than in the 0 mmHg group, but *Bax* levels did not show any significant difference. (*p* = 0.001 and 0.851, respectively; Fig. 3C). Subsequently, we focused on HSP90α encoded by *Hsp90aa1* and investigated the expression of HSP90α by immunocytochemistry, because *Hsp32*, known as an oxidative stress marker, is upregulated by compressive loading and oxidative stress leads to the release of HSP90α into the extracellular environment [30], [31]. Higher expression and nucleus translocation of HSP90α were observed in the 200 mmHg group when compared with the 0 mmHg group (Fig. 3D and 3E). Nucleus translocation of HSP90 occurs after cellular stress, and HSP90 tightly interacts with histones [32]. We decided to quantitatively evaluate HSP90α in the culture supernatants based on these observations.

### *Cd44*, *Has2*, and *Cox2* were upregulated by 6-h compressive loading but HA binding proteins and hyaluronidase gene expression were not

**Figure 4:**
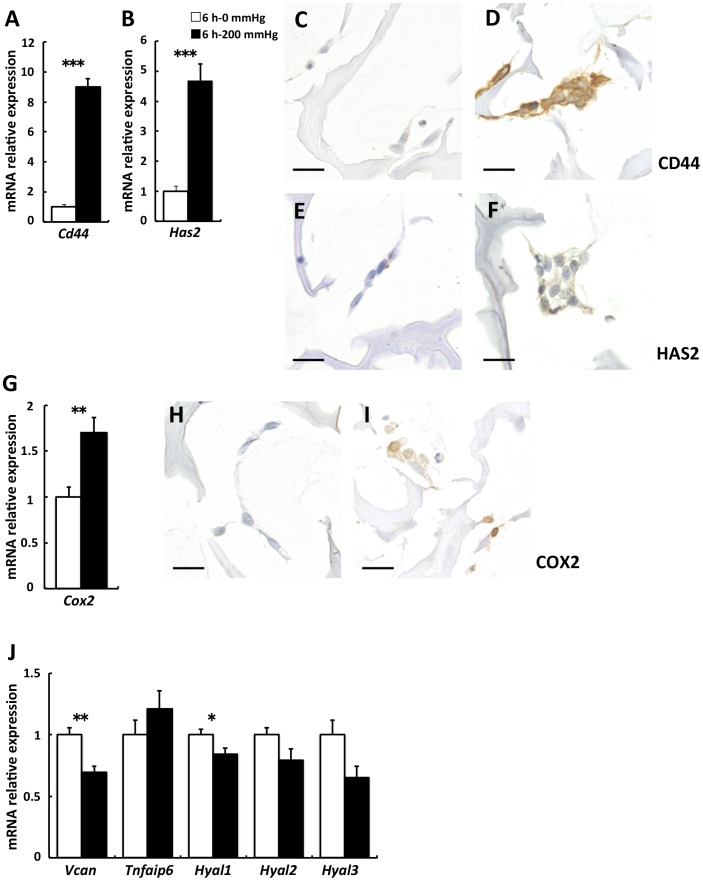
*Cd44* and *Has2* were upregulated by 6-h compressive loading, but HA binding proteins and hyaluronidase gene expression were not. Fibroblasts were seeded to collagen sponge and incubated for 24(□) or 200 mmHg (▪) compression for 6 h. Total mRNA was extracted after WST-1 assay, and mRNA expression was assessed using real-time RT-PCR. The expression of the target genes in the 6 h–200 mmHg group relative to the value in the 6 h–0 mmHg group was calculated by the comparative Ct method using the 18S ribosomal RNA gene as an internal control. The results are represented as the mean ± SEM (error bars) of five experiments. Statistical analysis was performed using the Student’s t test between the 0 mmHg group and the 200 mmHg group, and statistical significance was taken as *p*<0.05. A value of *p* was expressed as: *; *p*<0.05, **; *p*<0.01, and ***; *p*<0.001. A: *Cd44*. B: *Has2*. G: *Cox2*. J: *Vcan* and *Tnfaip6* are HA binding proteins. *Hyal1*, *2*, and *3* are HA degrading enzyme. C and D: Immunostaining for CD44. Representative sections of (C) the 6 h–0 mmHg group and (D) the 6 h–200 mmHg group. E and F: Immunostaining for HAS2. Representative sections of (E) the 6 h–0 mmHg group and (F) the 6 h–200 mmHg group. H and I: Immunostaining for COX2. Representative sections of (H) the 6 h–0 mmHg group and (I) the 6 h–200 mmHg group. Scale bars = 20 µm for all images.

A 9.0-fold increase occurred in *Cd44*, one of the adhesion molecules related to apoptosis *via* the disruption of adhesion [14], in the 200 mmHg group compared with the 0 mmHg group (*p*<0.001; Fig. 4A). Subsequently, we investigated *Has1*, *Has2*, and *Has3*, which are HA synthases, known as a primary ligand for CD44 [33]–[35]. A 4.6-fold increase occurred in *Has2* in the 200 mmHg group compared with the 0 mmHg group (*p*<0.001) (Fig. 4B). On the other hand, the levels of *Has1* and *Has3* were under the detection limit in both the groups. Subsequently, we investigated the expression of CD44 and HAS2 proteins by immunostaining because *Cd44* and *Has2* were significantly upregulated by compressive loading. Similar to gene expression, CD44 and HAS2 were upregulated by compressive loading in 3D cultured fibroblasts (Fig. 4C, 4D, 4E, and 4F). Based on these results, we decided to quantitatively evaluate HA synthesized by HAS2 in the culture supernatants. Furthermore, we investigated *Cox2* gene expression because CD44 and HA interaction upregulates COX2 expression [17]. *Cox2* was significantly upregulated by compressive loading (*p* = 0.007; Fig. 4G). Next, we investigated the expression of the COX2 protein by immunostaining based on the result for *Cox2* gene expression. Protein expression, as well as gene expression, for COX 2 was upregulated by compressive loading (Fig. 4H and 4I). Therefore, considering that PGE_2_ is a secretory substance downstream of COX2 [36], PGE_2_ would also be a possible molecular marker, that could be quantitatively evaluated in the culture supernatants.

In addition, for studying marker candidates besides HA and PGE_2_, we investigated the expression of *versican (Vcan), tumor necrosis factor alpha-induced protein 6* (*Tnfaip6*), *hyaluronidase 1 (Hyal1)*, *Hyal2*, and *Hyal3*, considering that the expression of HA binding proteins and degrading enzyme was upregulated along with an increase in *Has2* expression. However, the results indicated the expression of *Vcan* and *Hyal1* was significantly lower in the 200 mmHg group than in the 0 mmHg group (*Vcan*, *Tnfaip6*, *Hyal1*, *Hyal2*, and *Hyal3*; *p* = 0.003,  = 0.290,  = 0.045,  = 0.088, and  = 0.053, respectively; Fig. 4J).

### Secretions of HSP90α, HA and PGE_2_ into the cell culture medium were increased by 6-h compressive loading

**Figure 5:**
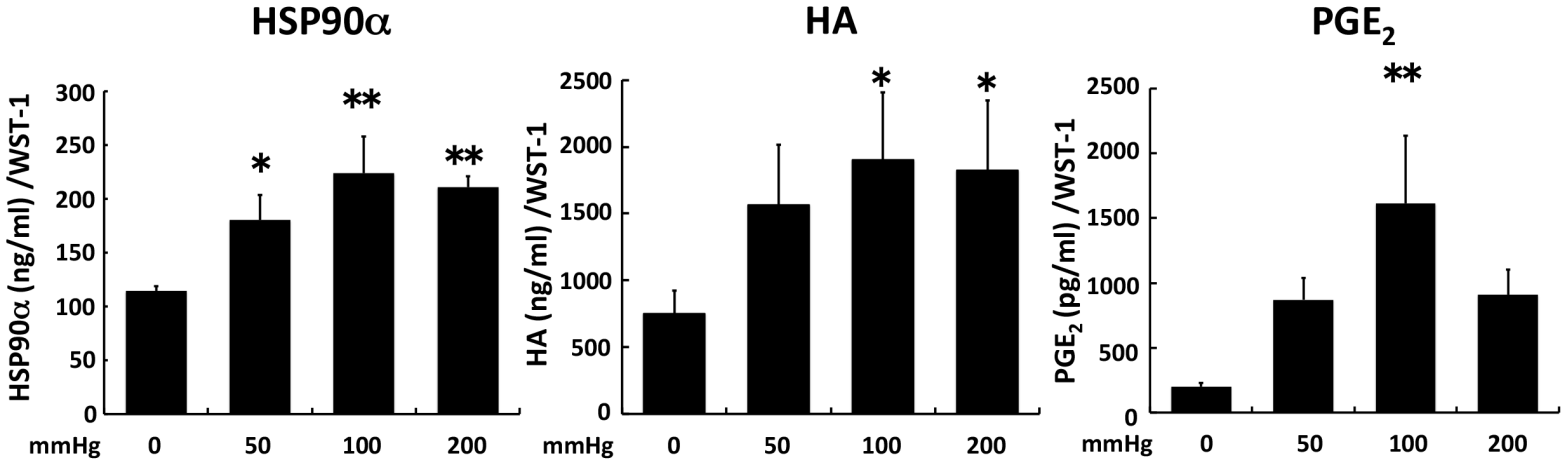
The secretions of HSP90α, HA, and PGE_2_ into cell culture medium were increased by compressive loading. Fibroblasts were seeded to collagen sponge and incubated for 24(A: HSP90α, B: HA, C: PGE_2_) was measured by ELISA. A value of concentration was normalized by WST-1 value. The results are represented as the mean ± SEM (error bars) of five experiments. Statistical analysis was performed using the Dunnett’s multiple test between non-loaded group and each of loaded group, and statistical significance was taken as *p*<0.05. A value of *p* was expressed as: *; *p*<0.05, **; *p*<0.01, and ***; *p*<0.001.

We then measured the concentration of HSP90α, HA, and PGE_2_ in the culture medium. The concentration of HSP90α was significantly higher in the 50, 100, and 200 mmHg groups than in the 0 mmHg group (*p* = 0.042,  = 0.002, and  = 0.004, respectively; Fig. 5A). The concentration of HA was also significantly higher in the 100 and 200 mmHg groups than in the 0 mmHg group (*p* = 0.014 and  = 0.021, respectively; Fig. 5B). On the other hand, the concentration of PGE_2_ was significantly higher only in the 100 mmHg group than in the 0 mmHg group (*p* = 0.004). An increase in PGE_2_ was observed in the 50 mmHg and the 200 mmHg groups compared with the 0 mmHg group (*p* = 0.177 and  = 0.149, respectively; Fig. 5C) but this difference was not statistically significant.

## Discussion

In the present study, we investigated candidates of molecular markers in order to predict delayed wound healing due to pressure focusing on cellular responses along with apoptosis triggered by the disruption of adhesion for the first time. Our results revealed that sustained compressive loading reduced the cell number and notably induced apoptosis in a time- and load-dependent manner. Furthermore, *Hsp90aa1, Cd44, Has2*, and *Cox2* were upregulated, and along with these upregulated genes, HSP90α, HA, and PGE_2_ were also increased by sustained compressive loading in fibroblasts. We noted the possibility of developing an assessment technology to predict delayed wound healing due to pressure, based on gene and protein expression and substances related to gene and protein expression analysis.

Wang and Thampatty [37] have reviewed many studies on the altered gene expression related to compressive loading in different cell types such as osteoblasts, chondrocytes, synovial cells, and periodontal ligament cells, which are exposed to compressive stimulation under physiological conditions. However, applying the previous results may not be appropriate for studying molecular markers induced by compressive stimulation in chronic wounds such as PUs because cellular responses are generally cell type dependent [37] and differs between sustained and cyclic manners for load application [15], [21].

Our results suggest that sustained compressive loading induced apoptosis and did not support apparent cell proliferation. Therefore, we studied the available molecular markers using our experimental system. In this system, inhibition of granulation occurred along with apoptosis. Our purpose was to study the cellular responses leading to molecular markers in the cells, during apoptosis in 3D cultured fibroblasts, which mimics the granulation tissue. Our results also show that apoptotic cell rate in the 6 h –200 mmHg was over 3 times higher than in the 6 h –0 mmHg. Although our system is single cell model, our results represent the effect of apoptosis observed in our model on delayed wound healing due to pressure at least in part. In fact, there are some animal models of chronic wounds [38]–[41]. However, these animal models employed two magnetic plates to dorsal skin for making PUs [38], [39] or treated with drugs to induce diabetic conditions before making full thickness wound [40], [41] to create the chronic wounds, therefore cannot be used for assessing the effect of pressure applied to the existing wounds on its healing process.

We found that apoptosis was induced and *Hsps* expression was higher in the 200 mmHg group compared with the 0 mmHg group. This result is consistent with previous reports. Recent studies have demonstrated that HSPs are upregulated by mechanical stress in periodontal ligament cells and gastric mucosa cells [10]–[12] and that apoptosis is induced by mechanical stress [20], [42], [43]. Sreedhar and Csermely [26] reviewed that the upregulation of HSPs occurs during the induction of apoptosis reported in many studies. Hence, apoptosis, upregulation of HSPs, and mechanical stress are closely related.

The increase in *Hsps*, *Bcl2*, and *Bax* gene expression observed in our study suggests that nonapoptotic surviving cells promote survival activity. The present study demonstrates that compressive loading leads to various biological stresses, which induces apoptosis, because various *Hsps* were significantly increased in the loaded fibroblasts. Upregulation of *Hsp32* indicates that oxidative stress is generated [44]. *Hsp47* and *Hspa5* are expressed during the stress in the endoplasmic reticulum [26], [45]. *Hsp60*, *Hsc70*, and *Hsp90aa1* encode chaperone proteins that repair damaged proteins and promote cell survival [26]. In addition, a correlation exists between ROS generation and the induction of HSPs [46], and the upregulation of BCL2 prevents mitochondrial ROS generation [47].

We observed remarkable upregulation of CD44 at both mRNA and protein levels by compressive loading. Upregulation of CD44 contributes to survival signals and promotes the resistance of apoptosis triggered by the disruption of adhesion [14], [48], [49]. Therefore, it is suggested that the induction of apoptosis in the present study has been caused by compression-induced disruption of adhesion.

The present study demonstrated that compressive loading increases the expression of HAS2 and that HA levels increase in fibroblasts. HAS2 synthesizes high molecular weight HA [50], which serves as a structural scaffold in the tissue [51]. HA has been shown to alter the physical properties of ECM [52], including hydration [53], diffusion [54], and viscoelasticity [55], [56]. HA associated with proteoglycan anchors to the cell surface *via* CD44 in the pericellular matrix [57]. HA also holds aggregates of proteoglycan [58] and forms huge complexes that provide a load-bearing function in ECM [59]. Increased HA in this system may therefore improve load-bearing function and scaffold for cellular adhesion. Takemura et al. [16] have reported that because of the elastic and hydrational properties of HA, an increase in HA by applying cyclic tensile stress to uterine cervical fibroblasts results in flexibility required during delivery. However, while upregulation of *Has2* and *Cd44* was observed by compressive loading, downregulation of *Vcan,* which encodes a core protein of proteoglycan, was also observed. Thus, it may not be sufficient to improve the load-bearing function and scaffold under sustained compressive loading. In previous study using tensile loading, Crockett et al. [15] have reported that the secretion of HA is increased in tendon fibroblasts, whereas the secretion of glycosaminoglycan becoming proteoglycan along with the core protein is not increased. This result is consistent with our result; however, in arterial smooth muscle cells using mechanical strain, the expression of protein and mRNA levels in versican was increased [60]. The difference in these results may occur as a result of the difference in the intensity and type of mechanical stress and cell type.

The present study also revealed upregulation of COX2 and PGE_2_ by compressive loading. Although an increase in PGE_2_ was observed in the 50 mmHg and the 200 mmHg groups when compared with the 0 mmHg group, a significant increase in PGE_2_ was observed only in the 100 mmHg group. Our observation that an increase in PGE_2_ does not occur in a load-dependent manner may suggest suppression of *Cox2* expression in the 200 mmHg group relative to that in the 100 mmHg group. As Misra et al. [17] have reported that fragmented HA suppresses *Cox2* expression, it is likely that HA was fragmented by ROS [61] in the 200 mmHg group and subsequently suppressed Cox2 expression. However, in our study the effect of compressive loading on the interaction of CD44 and HA and the occurrence of HA fragmentation has not been elucidated. Further studies are needed to establish these mechanisms.

The cellular responses reported here can be measured from the wound exudate because these factors are available in culture supernatants. Wound exudates may represent useful biomarkers in predicting the wound healing condition [8]. Furthermore, quantification of these markers using wound blotting [62] that has been developed in our laboratory would be applicable to a clinical setting.

We need to verify whether phenomena similar to those observed in our study also occur *in vivo* in animal experiments and in clinical patients. In this study, we investigated the *in vitro* cellular response of only 1 cell type, fibroblasts. The interaction of fibroblasts and inflammatory cells such as neutrophils and macrophages may alter cellular responses. Although our results show gene expression immediately after 6-h compressive loading, the expression change after release or repeated loading is unknown. Further studies are required to measure the concentration of matrix metalloproteinases, versican, HYAL1, and other factors, to improve the specificity of the assessment technology.

In this study, we investigated the cellular responses along with apoptosis triggered by sustained compressive loading-induced disruption of adhesion to predict delayed wound healing due to pressure. The results for the compressed samples demonstrated that apoptosis was induced in a load- and time-dependent manner; that *Hsp90aa1*, *Cd44*, *Has2*, and *Cox2* expression was upregulated; and that the concentrations of HSP90α, HA, and PGE_2_ increased in the culture medium. Therefore, we have newly introduced these candidate molecular markers for establishing a prediction method for delayed wound healing due to pressure.
