# Correction: Biological Responses of Three-Dimensional Cultured Fibroblasts by Sustained Compressive Loading Include Apoptosis and Survival Activity

**DOI:** 10.1371/journal.pone.0119308

**Published:** 2015-03-20

**Authors:** The PLOS ONE Staff

There is a missing paragraph under the sub-heading “Morphology and immunocytochemistry” of the “Materials and Methods” section. The fifth paragraph of this sub-section should read as follows.

To confirm nucleus translocation of HSP90α, HSP90α fluorescent immunostaining was performed using anti-HSP90α rabbit polyclonal antibody described above after semi boiling (diluted 1:50). Following methods are the same as vinculin. The translocation was observed in 3D (the X-Z and the Y-Z planes) as well as 2D (the X-Y plane) using a fluorescence microscope (BIOREVO, BZ-9000; Keyence, Osaka, Japan)

Second, there is some missing text prior to the penultimate sentence under the sub-heading “Stress- and apoptosis-related gene expression was stimulated by 6-h compressive loading” of the “Results” section. This sub-section should read as follows.

A significant increase in the expression of heat shock transcription factor 1 (Hsf1) and Hsf2 was observed in the 200 mmHg group compared with the 0 mmHg group (Hsf1 and Hsf2; p = 0.006 and = 0.004, respectively; Fig. 3A). Expression of these genes is induced by the disruption of adhesion [26], and HSF1 and HSF2 bind to the regulatory site of various Hsp genes. Following this, we investigated the influence of compressive loading on the gene expression of various HSPs, which are stress-responsive proteins against mechanical stress, elevated temperature, hypoxia, lowered pH, and reactive oxygen species (ROS) [27]. The expression of various Hsps was significantly higher in the 200 mmHg group than in the 0 mmHg group (Hsp32, Hsp40, Hsp47, Hsp60, Hspa5, Hsc70, and Hsp90aa1; p = 0.002, = 0.019, <0.001, = 0.024, = 0.033, <0.001, and <0.001, respectively; Fig. 3B). Upregulation of various Hsps indicates that stress responses by compressive loading occurred in fibroblasts. To examine the condition of nonapoptotic cells, we investigated the expression of antiapoptotic Bcl2 [28] and proapoptotic Bax [29]. The results indicated that Bcl2 levels were significantly higher in the 200 mmHg group than in the 0 mmHg group, but Bax levels did not show any significant difference. (p = 0.001 and 0.851, respectively; Fig. 3C). Subsequently, we focused on HSP90α encoded by Hsp90aa1 and investigated the expression of HSP90α by immunocytochemistry, because Hsp32, known as an oxidative stress marker, is upregulated by compressive loading and oxidative stress leads to the release of HSP90α into the extracellular environment [30], [31]. Higher expression and nucleus translocation of HSP90Α were observed in the 200 mmHg group when compared with the 0 mmHg group (Fig. 3D and 3E). To confirm nucleus translocation of HSP90α more precisely in the 200 mmHg group, we executed observation using a fluorescence microscope with full focus Function. A double stained nucleus with HSP90 and DAPI was focused in the X-Y plane. Three dimensional images with the Y-Z plane and the X-Z plane clearly demonstrated that the double staining was almost due to overlap of the distinct two green and blue spots, and HSP90 α was translocated into the nucleus (Fig. 3F). Nucleus translocation of HSP90 occurs after cellular stress, and HSP90 tightly interacts with histones [32]. We decided to quantitatively evaluate HSP90 α in the culture supernatants based on these observations.

Finally, the last sentence for the Fig. 3 legend is incorrect. The authors have provided a corrected version here.

**Fig 3:**
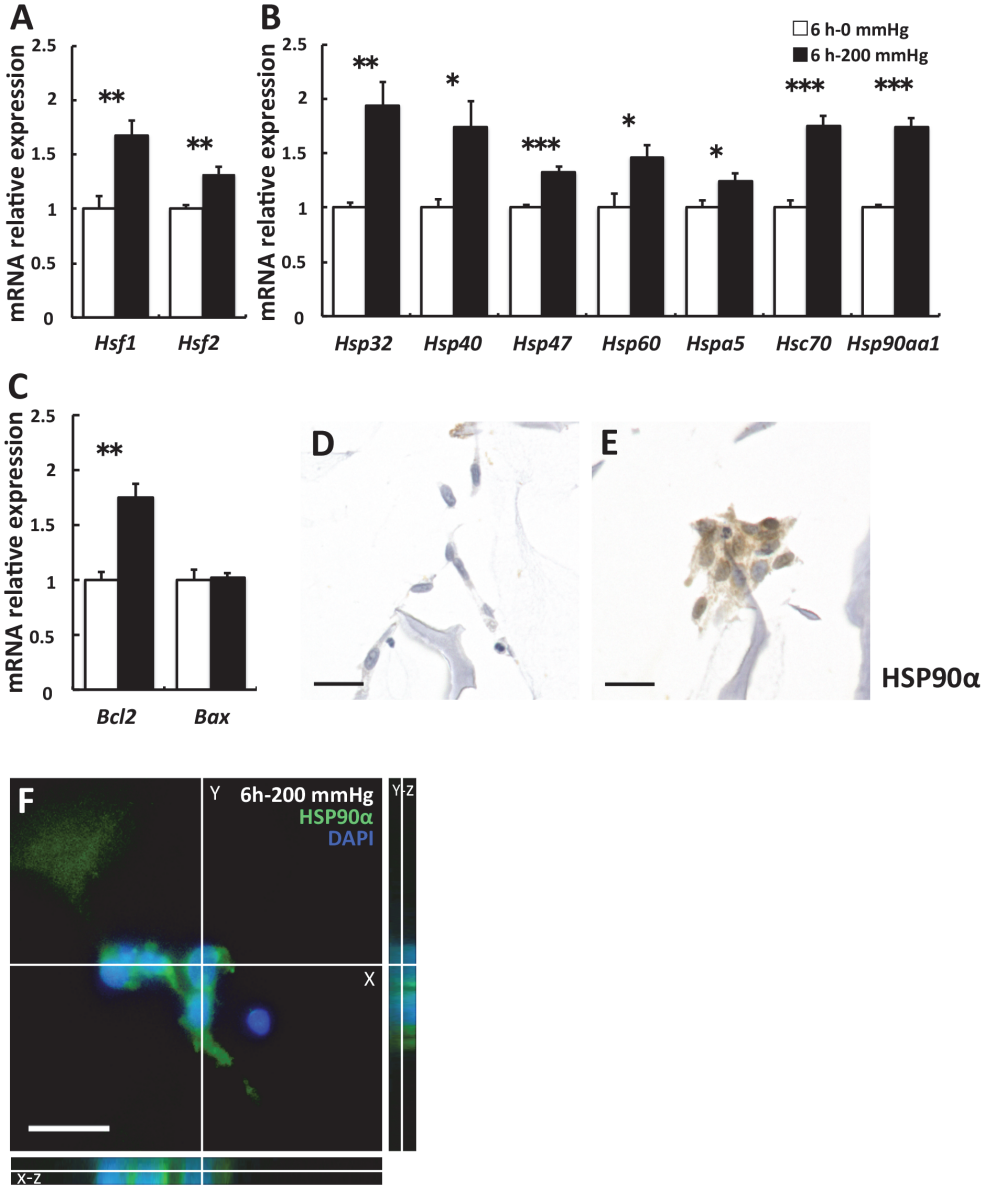
Stress- and apoptosis-related gene expression was stimulated by 6-h compressive loading. Fibroblasts were seeded to collagen sponge and incubated for 24 h. And then they were subjected to 0 mmHg (□) or 200 mmHg (■) compression for 6 h. Total mRNA was extracted after WST-1 assay, and mRNA expression was assessed using real-time RT-PCR. The expression of the target genes in the 6 h–200 mmHg group relative to the value in the 6 h–0 mmHg group was calculated by the comparative Ct method using the 18S ribosomal RNA gene as an internal control. The results are represented as the mean ± SEM (error bars) of five experiments. Statistical analysis was performed using the Student’s t test between the 0 mmHg group and the 200 mmHg group, and statistical significance was taken as p < 0.05. A value of p was expressed as: *; p < 0.05, **; p <0.01, and ***; p < 0.001. A: The transcription factors of various Hsps. B: various Hsps C: Bcl2 is an antiapoptotic gene, and Bax is a proapoptotic gene. D and E: Immunostaining for HSP90α. Representative sections of (D) the 6 h–0 mmHg group and (E) the 6 h–200 mmHg group. Higher expression and nucleus translocation of HSP90α was observed in the 200 mmHg group (E) when compared with the 0 mmHg group (D). F: Nucleus translocation of HSP90α in 3D images. Scale bars = 20 μm for all images.
